# Five regions, five retinopathy screening programmes: a systematic review of how Portugal addresses the challenge

**DOI:** 10.1186/s12913-021-06776-8

**Published:** 2021-07-30

**Authors:** Andreia Marisa Penso Pereira, Raul Manuel da Silva Laureano, Fernando Buarque de Lima Neto

**Affiliations:** 1 Instituto Universitário de Lisboa (ISCTE-IUL), ISTAR-IUL, Av. das Forças Armadas, 1649-026 Lisbon, Portugal; 2 Instituto Universitário de Lisboa (ISCTE-IUL), Business Research Unit (BRU-IUL) and ISTAR-IUL, Av. das Forças Armadas, 1649-026 Lisbon, Portugal; 3 Escola Politécnica, Computer Engineering (POLI/PPG-EC), Universidade de Pernambuco (UPE), Rua Benfica, 455 - Bloco ‘C’, Recife, 50720-001 Brazil

**Keywords:** Diabetic retinopathy, Population-based screening, Systematic review, Portuguese screenings

## Abstract

**Background:**

The implementation of a population-based screening programme for diabetic retinopathy involves several challenges, often leading to postponements and setbacks at high human and material costs. Thus, it is of the utmost importance to promote the sharing of experiences, successes, and difficulties. However, factors such as the existence of regional programmes, specificities of each country’s health systems, organisational and even linguistic barriers, make it difficult to create a solid framework that can be used as a basis for future projects.

**Methods:**

Web of Science and PubMed platforms were searched using appropriate key words. The review process resulted in 423 articles adherent to the search criteria, 28 of which were accepted and analysed. Web sites of all Portuguese governmental and non-governmental organisations, with a relevant role on the research topic, were inspected and 75 official documents were retrieved and analysed.

**Results:**

Since 2001, five regional screening programmes were gradually implemented under the guidelines of Portuguese General Health Department. However, complete population coverage was still not achieved. Among the main difficulties reported are the complex articulation between different levels of care providers, the low number of orthoptic technician in the national health system, the high burden that images grading, and treatment of positive cases represents for hospitals ophthalmology services, and low adherence rates. Yet, the comparison between strategies adopted in the different regions allowed the identification of potential solutions: hire orthoptic technician for primary health care units, eliminating the dependence of hospital professionals; use artificial intelligence algorithms for automatic retinographies grading, avoiding ophthalmologists overload; adoption of proximity strategies, as the use of portable retinographers, to promote adherence to screening.

**Conclusion:**

Access to diabetic retinopathy screening remains remarkably variable in Portugal and needs urgent attention. However, several characteristics of effective screening programmes were found in Portuguese screening programmes, what seems to point toward promising outcomes, especially if each other highlights are considered. The findings of this research could be very useful for the other countries with similar socio-political characteristics.

**Trial registration:**

PROSPERO registration ID CRD42020200115.

**Supplementary Information:**

The online version contains supplementary material available at 10.1186/s12913-021-06776-8.

## Contributions

- This study contributes to the assemblage of knowledge in the field of diabetic retinopathy screenings, providing the first systematic review of the Portuguese experience.
- The study also details the main diabetic retinopathy screening implementation problems. It points out the possible solutions for operational planning of future screenings, the improvement possible for the existing ones, and put forward a framework to comparative analyses.
- This study highlights the importance of adequate governmental funding, national guidelines that precise the role of the different intervenient, and of politic measures that guarantee the involvement of all parts.

## Background

Diabetes Mellitus (DM) is a chronic metabolic disease and one of the most prevalent diseases worldwide [1–4]. DM can cause macro and microvascular complications, including diabetic retinopathy (DR) [5–7]. DR occurs when blood vessels in the light-sensitive region of the eye, the retina, leak or become blocked, due to prolonged high blood glucose levels [8, 9]. DR is the most common cause of vision loss in people with diabetes [7, 10] and globally is the leading cause of visual impairment and blindness among working age population [11–13]. However, DR can be prevented or delayed by timely diagnosis and management of diabetes [14, 15], and blindness can also be prevented or delayed by regular eye screening and appropriate treatment [16, 17].

Nonetheless, although extremely important, the implementation of a population-based DR screening, requires the intervention of many stakeholders (government, hospitals, primary health care units) and involves numerous challenges, which often lead to unexpected setbacks at high human and material costs [18, 19]. Thus, the share of knowledge and experiences between countries is of recognised utility, and there is a permeant need for a solid framework, that can be used as a basis for future projects [5, 18, 20]. However, the desirable interchange is not easy to accomplish. In fact, different countries often have different health systems, which makes it difficult to understand and categorise procedures [5, 21], screening programmes may be implemented at a national, regional, or local level, resulting in sparse information at a national level, and there are organisational and even linguistic barriers, that complicate the process [18, 20].

In this context, this study intends to answer the following research question: How is the population-based DR screening programme conducted in Portugal? And, consequently, to contribute to the assemblage of knowledge in the field of DR screening, providing a systematic scientific and technical literature review of the Portuguese experience, which can be used to plan future programmes or implement improvements in the existing ones.

The strategic planning of a DR screening requires a deep knowledge to be successful [20]. So, in this paper five key questions are addressed, namely: i) What are the general guidelines of the screening programmes in Portugal? ii) How did each region implement the screening? iii) What are the main metrics used to measure the results of each screening programme and how did DR Screening results evolved through time? iv) What are the main problems reported when implementing DR screening programmes and how can eventual risks be mitigated?

By analysing the accepted 28 scientific peer-reviewed articles and 75 technical documents from government (e.g., [22, 23]) and non-governmental organisations (e.g., [24, 25]), five Portuguese regional DR screening programmes, within the context of the National Health System (SNS), allowed the identification of the advantages and weaknesses of each regional strategy and are discussed in the light of documented international experiences. Most of the available studies about DR screening are cost-effectiveness analyses (e.g., [26, 27]), or are focused on very specific aspects of the process (for example automatic reading grading [19, 28]). However, the overall screening strategy is rarely well described [16] and normally only the unilateral point of view of one type of stakeholder is explored, e.g., diabetics [29], health professionals [30], primary health care units [30], hospitals [31] and government [27]. As opposed to that, in this review we specifically tried to identify alternative screening strategies and assess the challenges faced by the different levels of health care providers, producing a synthesis of the evidence available in the literature.

This work is organised as follows. The first section concerns the adopted methodology and literature selection. Then, the general guidelines of the screening programme in Portugal, the differences between regional protocols, the indicators used to measure screening results, the quality evaluation, and the main problems reported in implementing DR screening programmes, are analysed. Finally, the implications of the different scenarios are examined considering the best national and international practices.

## Methods

### Search for studies

We performed a systematic review according to the Preferred Reporting Items for Systematic Reviews and Meta-Analysis (PRISMA) checklist [32] (see Additional file 1).

For the scientific review, Web of Sciences (www.webofknowledge.com) and PubMed (https://www.ncbi.nlm.nih.gov/pubmed/) databases were searched. The selection of scientific databases was based on their scope and their wide range of publications in the field of interest [33, 34]. Moreover, these databases are frequently used in other researches [33].

The search was performed according to the following query: ((“Diabetic retinopathy” or “DR” or “diabetic vision lost” or “diabetic complication*”) and (“screening” or “preventive public policy*” or “preventive eye exam” or “early diagnosis” or “retinography”) and (“population based” or “mass”)).

The query was applied to the topic (title, abstract and keywords) field, for the period 2009–2020 and only considering articles written in English or Portuguese languages. The time constraint was imposed because, in Portugal, there is no truly population-based DR screening, prior to the year 2009. The linguistic restriction is due to the very purpose of this systematic review – to analyse the screening of DR in Portugal – and, to the fact, that English is nowadays the universal language in the scientific world.

Technical documents were retrieved from the web sites of all Portuguese governmental and non-governmental organisations, with a relevant role on DR Screening (see Additional file 2). Governmental organisations were selected based on their mission and in the organisational chart of the National Health System. Non-governmental organisations were identified through references of papers and official documents.

### Inclusion and exclusion criteria

Inclusion and exclusion criteria were applied to select the relevant set of articles to be reviewed.

For the scientific review, were included studies published in pear review journals, referring to the DR screening programme in Portugal. Articles that were focused on interventions, clinical rehearsals, research with methodological deficiencies, and duplicate work were excluded. Four hundred and twenty-three articles were retrieved from Web of Sciences and PubMed databases. A preliminary review process was applied according to the following steps: 1) exclusion duplicate articles; 2) evaluation of scientific articles according to abstracts excluding those focused-on interventions, clinical rehearsals, research with methodological deficiencies. This preliminary evaluation resulted on the exclusion of 64 articles.

For the technical review were included only official documents, available on the institution web site, and referring to DR screening programme. Excluded documents were those that are not dully substantiated and duplicate work. Regarding technical documents, 1 hundred and 75 were retrieved from the web sites of all Portuguese governmental and non-governmental organisations (listed in Additional file 2), with a relevant role on DR Screening. After the preliminary evaluation, 97 official documents were selected and analysed.

**Fig. 1:**
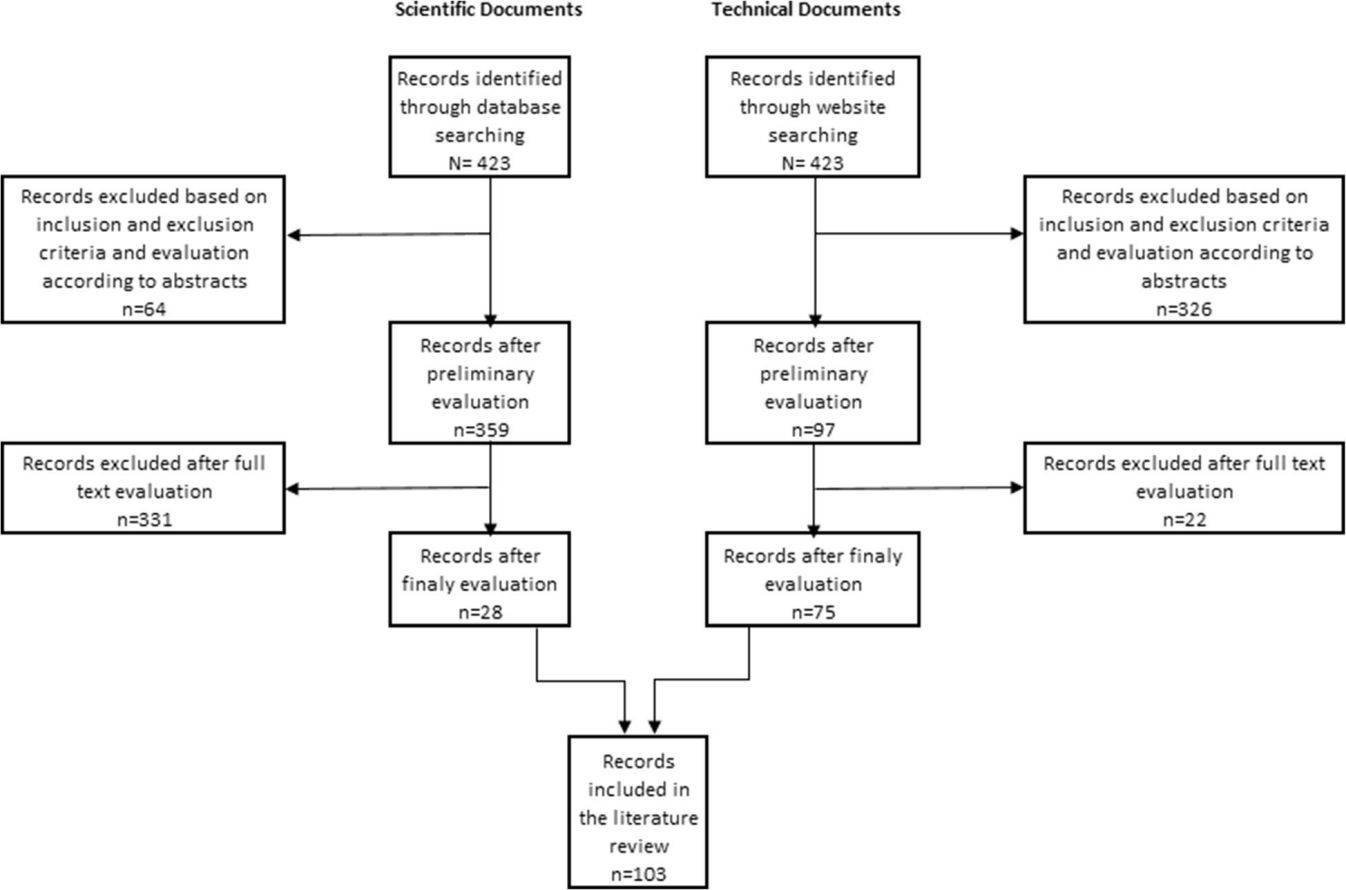
Flow chart of scientific/technical documents selection process

Finally, all the selected documents were submitted to a critical full document evaluation, what allowed to exclude articles that did not mentioned the Portuguese DR screening programmes, scientific or technical documents with methodological deficiencies and the ones not dully substantiated. Two experts of the Portuguese North Region Health Administration validated both selection procedures. After the selection process, 28 articles and 25 official documents remained. Figure 1 illustrates the selection process.

#### Articles and documents analyses procedure

To facilitate the documents analysis, they were organised in different categories. The documents classification was carried out by two of the three authors of this systematic review. The third researcher was called to break the tie, whenever there was no agreement between the first two.

Scientific documents were divided in three categories: i) DR Incidence / prevalence studies or studies focus on DR characteristics, such as risk factors, natural history and, progression **(**10 papers); ii) Machine learning algorithms for images grading (10 papers); and iii) Screening strategies (five papers). Three papers were classified in both 1 and 2 categories. Additionally, to access the quality of the scientific articles eight quality items were considered (Table 1) and graded according with the following rule: Yes(Y) = 1; No(N) = 0; Partially(P) = 0.5. The marking of the selected papers in each of the quality criteria is available in Additional file 3.

**Table 1:** Quality Criteria Items

ID	Quality Criteria
PQ02	Are the details of the screening protocol well described?
PQ03	Are the sources reliable?
PQ04	Is the methodology used rigorous and replicable?
PQ03	The geographical area covered and the institution responsible for the screening are well identified?
PQ04	Are the indicators used to measure screening results well described?
PQ05	Does it identify the problems that affect the implementation of population-based screening programs?
PQ06	Does it identify the constraints that affect the implementation of population-based screening programs?
PQ07	Does it identify solutions and best practices from national or international experiences concerning population-based DR screening?
PQ08	Does it objectively describe the evolution of DR screening over a considerably large period?

Official technical documents were classified as documents of national scope (24) or documents of regional scope (51). Regional documents were distributed by North (11), Central (10), Lisbon and Tagus Valley (10), Alentejo (10) and Algarve (10) regions.

## Results

### General guidelines of the Portuguese screening programme

In 1998, the Portuguese General Health Department (DGS) has established the first guidelines for DR population-based screening programmes. Non-mydriatic Chamber Fundus Photography (colour retinography) was the recommended screening method, due to its high sensitivity and specificity (92 and 90% respectively), and because this method can be performed by trained paramedical personnel and later sent for ophthalmologist analyses. Annual screenings were recommended for diabetics after puberty [22]. The costs of the screening and treatment for DR are completely covered by the government. Only indirect costs, as transportation to the screening or treatment facility, are supported by the diabetics [22]. Regional Health Administrations (ARS) have the responsibility of operationalise population-based screening programmes. In Portugal there are five ARS (ARS North, Central, Lisbon and Tagus Valley, Alentejo and Algarve). So, since 2001, the ARS began the implementation of screening strategies under DGS guidelines [35–39]. None the less, the guidelines were vague in what concerns to major operationalisation aspects as what services and health staff should be involved and which are their responsibilities, where the screening test should take place, who identifies and convokes the diabetic populations, etc. Therefore, the strategies adopted by each ARS are significantly different [35–39]. Regarding positive cases, all the ARS mention referral for a hospital ophthalmology consultation, where a diagnosis is made and a treatment plan appropriate to the stage of the disease is established. However, despite the treatment being guaranteed, there were no guidelines for its standardisation at national level. The definition of a positive case itself, that is, requiring referral for ophthalmology consultation, was not uniform in all regions [35–39].

**Fig. 2:**
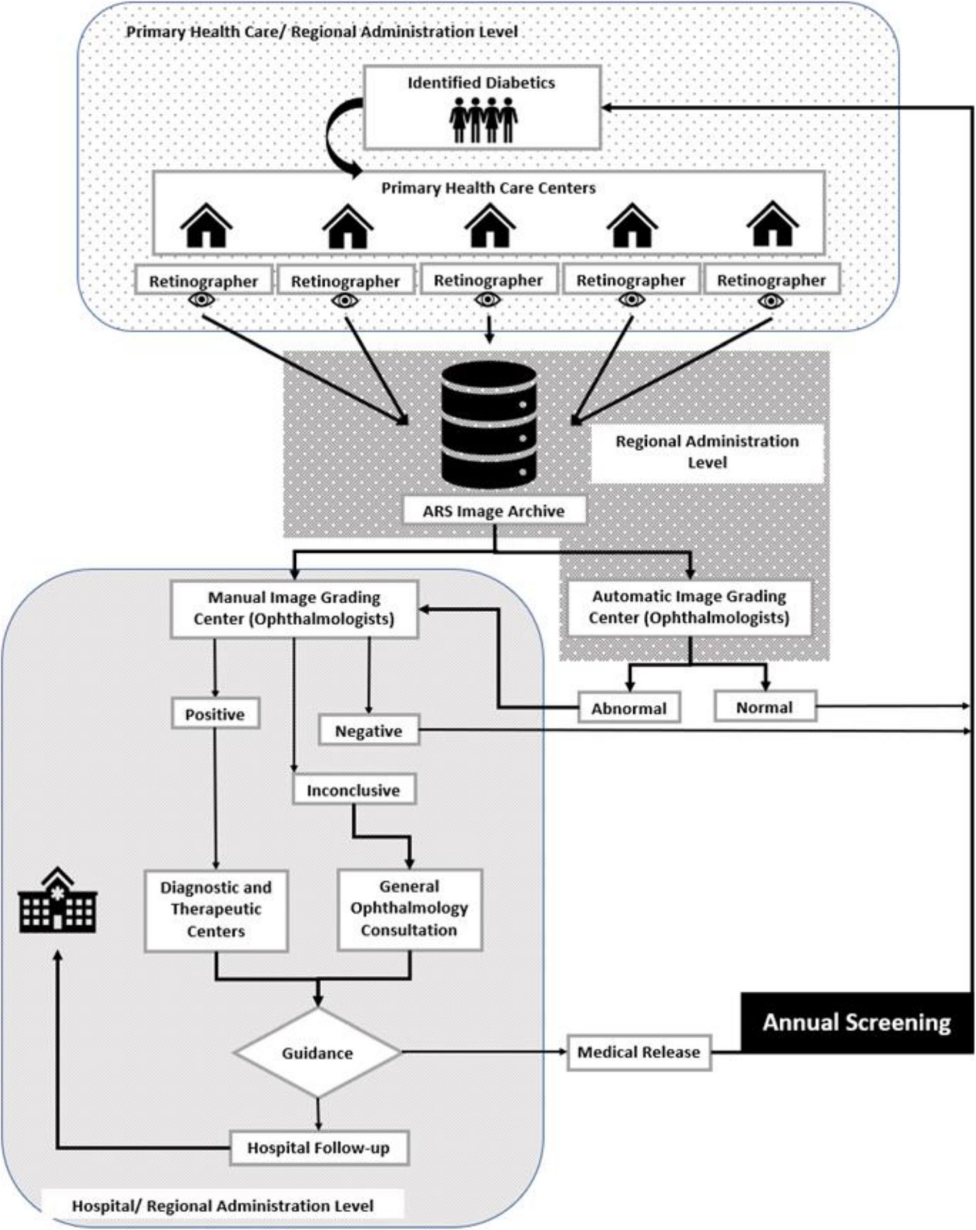
DGS Screening Flow Chart. Adapted from [41]

In 2018, DGS issued new and more detailed, guidelines for the organisation of regional screening programmes [40], proposing a flow chart for the screening process (Fig. 2).

DR Grading and the definition of Positive and Negative, were also clarified and normalised, trough the referral guidelines summarised in Table 2 [40].

**Table 2:** DGS 2018 referral guidelines. Adapted from [40]

**Diabetic retinopathy screening result**		**Referral**
R0	No disease visible	Repeats screening after a year
R1	Mild No Proliferative DR (NPDR)	Repeats screening after a year
R2	Moderate NPDR	CDTI 1, 2, 3 RD ophthalmologic consultation in a two-month period
R3	Severe NPDR	CDTI 2, 3 RD ophthalmologic consultation in 1 month period
	Proliferative DR (PDR)	
M1	Diabetic Macular Edema (DME)	
V1	Hight risk PDR, vitreous haemorrhage, or tractional retinal detachment	CDTI 3 RD ophthalmologic consultation in a 15-day period
ICN	Inconclusive or comorbidities	General ophthalmology consultation
**Treatment follow-uo**		
P0	Stable LASER	Repeats after a year
P1	Insufficient LASER	CDTI 1 (Thermic LASER)

Table 3 summarises the DGS recommended procedures and treatments for the different stages of DR [40].

**Table 3:** DGS 2018 treatment guidelines. Adapted from [40]

Diabetic retinopathy stage	Procedure and treatment
No disease visible or Mild NPDR	DR screening
NPDR moderate or severe	DR ophthalmologic consultation Optical coherence tomography (OCT)
NPDR with DME focal or multifocal or PDR without DME	Fluorescein angiography (FA) and OCT Laser therapy
PDR with MDE Diffuse DME	FA + OCT Combined DR therapy: Laser + Intravitreal injection of anti-vascular endothelial growth and/or long-acting corticosteroids
Advanced PDR with: - Vitreous or sub hyaloid haemorrhage - Retinal detachment - Neovascular glaucoma - Chronic DME with no response to treatment or refractory	DR chirurgic therapy: vitrectomy Combined DR therapy: FA + OCT + Laser + Intravitreal injection of anti-vascular endothelial growth and/or long-acting corticosteroids Corticosteroids extended-release injectable devices

### Regional DR screening protocols

At the North Regional Health Administration (ARSN), the DR Screening Programme began in 2009, and has been gradually implemented in the following years. In 2009, ARSN, developed exhaustive proceedings, documentation, and protocols, which have been subsequently expanded and adjusted [39]. In this region, retinographies are performed in Primary Health Centres. However, there are no fixed retinographers in health facilities. The equipment remains in mobile units, moving from health centre to health centre, according to prior established schedule [39]. Primary Health Centres are responsible for identifying and convening the diabetic population and retinographies are performed by orthoptics technicians. However, there are no orthoptics technicians dedicated solely to the screening programme. Those professionals are provided by local hospitals, and usually accumulate the functions inherent to the screening programme, with the functions they perform regularly in the hospitals. After the retinographies are performed they are analysed and graded by ophthalmologists [39]. ARSN is conducting a research aiming the introduction of automatic image reading software in DR screening programme, however, this technology is still experimental [42]. After the grading, positive cases are referred to the hospital for treatment. Since the beginning of the screening programme, ARSN uses the International Clinical Classification System, which categorises DR severity in 5 levels, including 3 stages of low risk: none, mild, and moderate NPDR, a fourth stage of severe NPDR, and a fifth stage of PDR, in the presence or absence of DME, which is graded separately (as recommended by the 2018 DGS guidelines – Table 1) [39, 40]. The ARSN uses a specific software to support the screening programme (SIIMAScreenings) [23].

At the Portuguese Central Region Health Administration (ARSC), the DR Screening Programme is running since 2001 [37]. As in the North Region, the screening method and the target population follow the 1998 DGS guidelines [37]. Until 2011, the screening protocol was similar to the one implemented at ARSN. However, in that year, was introduced the use of an automatic image reading software (RetmarkerSR) in conjunction with the traditional human analysis and grading. This software allows the detection of RD lesions such as DME and small haemorrhages in retinal photographs, through a method based on image processing algorithms [37]. Two of the selected papers focus on the performance of this particular software revealing a sensitivity of 99.76% and a specificity of 99.49% [43, 44]. The grading scale used in ARS Centro, is different from the 2018 DGS guidelines. The scale includes 5 different classification levels: NC – not classifiable; R0 – no DR lesions; RL - NPDR without maculopathy; M - maculopathy; and RP - PDR. In ARS Centro, referable diabetic retinopathy, was defined for all patients graded as NPDR, PDR, or M [43]. Another particularity of ARSC Screening is that there is no software application to support the screening programme. The data are requested by the ARSC to each of the Primary Health Centre Clusters (ACES) and compiled into Excel sheets [37].

Lisbon and Tagus Valley Regional Health Administration (ARSLVT) and the Association for the Protection of Diabetics of Portugal (APDP) signed a cooperation protocol in 2009, for DR screening [38]. It was the beginning of Diabetic Retinopathy Screening Service for Lisbon and Tagus Valley (RETINODIAB), commissioned and driven by APDP and supported by ARSLVT. The RETINODIAB follows the 1998 DGS norms in terms of screening test and target population [45]. In 2016, the ARSLVT implemented their own pilot screening in four ACES. Accordingly, with this established protocol, the retinographies are performed by orthoptists, in the ACES, and automatically analysed and graded by a software - “Retmarker”. When classified by the software as “necessary human reading”, they are sent for ophthalmologists’ analysis. The results of these readings are made available to the family doctor by means of a computerised screening platform. As in ARSN, the DR grading scale used is according to the 2018 DGS guidelines [45]. Positive (except Mild NPDR) and inconclusive cases are referenced to hospital ophthalmology services [38]. Nowadays, ARSLVT, extended this new screening programme, and APDP, RETINODIAB, is still a complementary response, continuing to cover 7 of the 15 ACES [38]. In ARSLVT, the screening programme is computer-supported by SIIMAScreenings in 4 ACES and by the APDP system in 7 [38]. An internal recruitment process for orthoptists for Primary Health Care has begun in 2017 [38].

At Alentejo Regional Health Administration (ARS Alentejo), there is no standardised screening strategy. In fact, there are three different screenings. The DR screening managed by ARS Alentejo, which began in 2011 and follows the 1998 DGS guidelines in terms of method and target population, is implemented in one ACES. The retinographies are performed by orthoptic technicians provided by hospitals and uses SIIMAScreenings as screening computer-system [35]. In a second ACES, family doctors refer patients with diabetes to perform the retinography in the hospital, so the data related to this ACES are not introduced in the screening platform. And, in a third area the screening is carried out in partnership with APDP [35].

In March 2013, the Algarve Regional Health Administration (ARS Algarve) began the implementation of a population-based screening for all diabetics in the region [36].

The screening test is performed by the two Hospitals in Algarve, in the ophthalmology departments. The articulation between ARS Algarve and the hospitals is performed through protocols and annual contracting. Screening monitoring is computer-supported [36].

During the year 2014, hospitals were reticent about the renewal of the screening protocol due to the reduce installed capacity. So, ARS Algarve proposed to limit the screening, in this period, to the “new cases” diagnosed during 2013 and 2014 what was accomplished by the end of the year [36]. In 2015 and 2016, the screening was resumed in a normal way. However, in 2017 and 2018, the screening did not take place. In that year’s activities report, ARS Algarve claims that, although the normal procedures for the renewal of the programme were carried out, there was any hospital response and that, despite having taken countless efforts to develop a screening programme less dependent on hospital capacity (similar to those existing in the North, Centre and part of Lisbon and Vale do Tejo), this was not possible due to numerous procedural constraints [36].

The analysis of the technical documentation of the five ARS, showed that there are considerable differences between the implemented screening programmes (Table 4) [35–39]:

- The screening location varies according to the region: in ARSN and ARSC there are portable retinographers which, in turn, are allocated to the Primary Health Centres of the region [37, 39]; at ARSLVT there are fixed retinographers in Primary Health Care units [38], and in ARS Algarve all screening phases are performed by hospital ophthalmology services.
- If, in some ARS, retinographies are performed by hospital orthoptic technicians, which accumulate the functions in the hospital with the DR screening [39], other (ARSLVT) are hiring optometrists for primary health care units [38]. Although this solution seems simple and effective on eliminating the dependence of available hospital technicians, it is not easy to implement, mostly due to the lack of consensus on the competence of optometrists to perform retinographies. In fact, there are substantial differences in the training of the two types of professionals: orthoptic technicians are qualified to detect vision abnormalities and ocular motility disorders. Therefore, the orthoptic technician is active in diagnosis, therapy, and rehabilitation; on the other hand, optometrists are the professionals that, through examination of the eye, diagnoses refractive errors and prescribes appropriate lenses and/or exercises, without the need for drug or surgical treatments [40, 46]. However, there are several countries in which the retinographies are carried out by professionals other than orthotics technicians or optometrists, for example, primary care physicians or nurses [17, 47], but in Portugal those options were never considered.
- In the ARSC and in part of the ARLVT region, artificial intelligence software is implemented for automatic retinographies grading [43–45]. Several studies state its acceptable sensitivity and specificity levels and its effectiveness to reduce ophthalmology services burden [48–50].

**Table 4:** Screening Protocol

	North	Central	LVT	Alentejo	Algarve
**Screening method - colour fundus photography**	Yes	Yes	Yes	Yes	Yes
**Electronic transfer of images**	Yes	Yes	Yes	Yes	Yes
**Retinografers location**	Portable	Portable s	Non-portable - ACES	U	Hospital
**Pupil dilatation**	No	No	No	No	No
**Calls to the target population through postal invitations**	Yes	Yes	Yes	Yes	Yes
**Who performs the photography**	Orthoptic technicians provided by hospitals	Orthoptic technicians provided by hospitals	Primary Health Centres orthoptists	U	Hospital Orthoptic technicians
**Software for automatic readings**	No	Yes	Yes	No	No
**Camara Device**	Non-mydriatic camera, CR-2 Digital Retinal Camera (Canon)	Nonmydriatic cameras – Canon CR6-45NM with a Sony DXC-950P 3CCD colour video camera	Non-mydriatic camera, CR-2 Digital Retinal Camera (Canon)	U	U
**Screening test procedure**	Retinography of both retinal fields, both with 45° field, one focusing on the macula and the other on the optic nerve	Retinography of both retinal fields, both with 45° field, one focusing on the macula and the other on the optic nerve. When impossible to obtain an image with minimum quality is performed an iatrogenic pupil dilation with a topical mydriatic.	Retinography of both retinal fields, both with 45° field, one focusing on the macula and the other on the optic nerve	U	U

*U* Unavailable

The new DGS directives substantiate an important attempt to guarantee quality, equity of access and standardisation of screening at national level [40]. However, the analysis of the latest activity reports of the ARS (2018), clearly shows that, so far, the new guidelines have not produced many effects at the regional level. Thus, while some ARS established procedures perfectly framed with the guidelines now issued, there are others, in which the so-called population-based screening programmes fall far short of the requirements that the denomination, and the current national guidelines, require [35–39].

### Main indicators and screening results

The analysis of the official reports of the Portuguese institutions directly involved in the implementation of the DR screenings allowed to determine a set of common indicators, used to monitor the process and the results of the screening programmes.

However, the number of available indicators is very small, reflecting only the concern with the coverage of the screening [35–39]. No indicators inherent to the quality of the process were found in any of the five ARS. In rare cases, references to the evolution of the number of positive DR cases were found, which, however, were discarded due to important inconsistencies in the concept of “positive case” itself. Still, it was found that most ARS collect and report the following indicators [35–39]:

$$ Geographic\ coverage=\frac{Number\ of\ ACES\  on\  the\ programme}{Total\ ACES\ of\ the\ Region} $$

$$ Adherence\ rate=\frac{Number\ of\ retinographies}{Number\ of\ invitations} $$

$$ Population\ coverge=\frac{Number\ of\ invitations}{Number\ of\ identified\ diabetics} $$

$$ Screened\ population=\frac{Number\ of\ retinographies}{Number\ of\ identified\ diabetics} $$

As previously mentioned, generally, the indicators are calculated by the ARS, although the data are obtained directly through an operating system dedicated to screening, or indirectly, through requests to the primary health units, or associations involved (APDP, hospitals) Of course, when the second case occurs, less reliability of the data is expected, since it is common for different entities to follow different criteria for extracting and pre-processing the information.

But, in addition to this issue, there are other inconsistencies in the calculation of the indicators [35–39]:

1. First, as we have seen, there are several ARSs (part of ARS LVT, ARS Alentejo and ARS Algarve) where screening is still conducted, in whole or in part, by other institutions, leaving the question of whether it is truly a population-based screening. Normally, the ACES where this happens are counted as being covered by a screening programme, but, at the risk of, in some cases, providing only an opportunistic screening to registered diabetics. This inconsistence will affect the “Geographic Coverage” indicator.
2. The variable “number of identified diabetics” is also likely to introduce some bias in the analysis of the results. In reality, not all identified diabetics are convolved into screening. According to the DGS guidelines, family doctors should remove from the list the subjects who are unable to remain seated, those who underwent a retinography less than a year ago and those who are blind. Thus, it is important to distinguish whether the ARS account for the initial number of identified diabetics, or that obtained after the purging of the initial listings. The “Population coverage” and “Screened population” indicators could be affected by these decisions.
3. The variable “Number of invitations” is also not easy to measure. In fact, so far, none of the ARS has managed to strictly comply with the 12-month interval between screenings. Therefore, at the time of the change of civil year, there are several locations with the annual screening still in progress. Thus, these questions arise: is it effective only to consider invitation letters in places where the screening has already been completed? All invitation letters sent should be considered, even if, in some cases diabetics have not yet had the opportunity to adhere to the screening, simply because, the screening was scheduled for a date later than the present moment? The assumptions in each case are not clear and may condition the comparison of adherence rates between ARS. The “Population coverage” and the “Adherence rate” are affected by this bias.

Despite the constraints mentioned previously, the following Tables 5, 6, and 7 show the available indicators, in each of the five ARS. Due to the scarcity of information in some of the ARS, it was decided to present the results only for 2015 and 2017 (years in which more comprehensive information was obtained) [39]. The variable “Number of retinographs performed” was the only one that allowed an evolutionary analysis, which is presented in Table 7 [35–39].

**Table 5:** 2015 Results

	North	Central	LVT	Alentejo	Algarve	Total
**ACES on the programme (a)**	17	8	11	4	2	42
**Total ACES (b)**	24	8	15	4	3	54
**Geographic coverage (a)/(b)**	70.8%	100.0%	73.3%	100.0%	66.7%	77.8%
**Identified diabetics (c)**	277,706	142,008	183,958	47,221	U	674,537
**Number of invitations (d)**	75,767	U	57,049	3501	23,404	159,721
**Number of retinographies (e)**	45,119	19,792	35,602	3477	16,491	120,481
**Percentage of ungradable images**	3,2%	3,5%	3,7%	U	U	U
**Adherence rate = (e)/(d)**	59.5%	U	62.4%	99.3%	70.5%	75.4%
**Population coverage = (d)/ (c)**	27.3%	U	31,0%	7,4%	U	23.7%
**Screened population = (e)/ (c)**	16.2%	13.9%	19.4%	7.4%	U	17.9%

*U* Unavailable

**Table 6:** 2017 Results

	North	Central	LVT	Alentejo	Algarve
**Geographic coverage 2017**	75%	63%	100%	50%	0%
**Adherence rate 2017**	60%	U	52%	91%	NA
**Screened population 2017**	35%	9%	29%	6%	0

*U* Unavailable, *NA* Not Applied

**Table 7:** Evolution of the number of retinographies

ARS	2009	2010	2011	2012	2013	2014	2015	2016	2017
North	791	8839	39,006	49,354	57,385	47,454	45,121	68,309	105,462
Central	14,760	15,271	15,258	18,496	11,856	13,235	19,792	U	U
LTV	3131	13,867	23,221	24,819	28,272	25,853	28,562	35,602	74,744
Alentejo	U	2761	2872	2512	1668	7573	3477	7144	2799
Algarve	10,907	9395	13,580	7937	16,103	1420	14,491	U	0

*U* Unavailable

Despite several setbacks in all regions, the number of screenings has been increasing since 2009. In 2015, a total of 113,443 retinographies were taken, 19% more than in the same period of 2014 (Table 7). However, access to diabetic retinopathy screening is still remarkably variable in Portugal and needs urgent attention. Population coverage, in 2017 varies from 0% in ARS Algarve to 100% in ARSLVT (Table 6) [35–39].

## Discussion

Retinopathy screening involves several interfaces where communication can be problematic (family doctor, patients, optometrists, regional screening teams, hospitals, ophthalmologists) [20, 21, 51, 52]. A major effort is necessary to understand and coordinate this complex system with dynamic interactions of different agents (stakeholders, providers, professionals, and individuals), and where change in any one element can alter the context for all other elements [20]. So, national guidelines should precise the role of the different intervenient, and politic measures should be created to guarantee the involvement of all parts.

According to official documents, another of the major problems for the sustainability of Portuguese screening programmes is the lack of orthoptic technicians in the SNS. ARS where retinographies are performed by hospital orthoptic technicians, which accumulate the functions in the hospital with the DR screening, are dealing with permanent difficulties to ensure the full coverage of the programme [37, 39]. In fact, this situation led to interruption of screenings in sites that had already started and can represent a major sustainability problem [39]. In addition, some ARS reported difficulties in ensuring the first hospital visit within 30 days of the diagnosis of DR [39].

To truly understand these problems is important to know the hospitals point of view. Opportunely, one of the selected studies took place at the Hospital Centre of Oporto (CHP), and provides the perspective of this hospital ophthalmology services [31]. The CHP is the reference hospital for 2 ARSN’s ACES, which together represent about 293,900 inhabitants and 24,902 diabetics (data for 2016) [31]. An important finding that emerges from this research is that the screening programme is referencing to ophthalmologic consultation, patients who are already being followed in hospital services. ARSN screening protocol recommends the exclusion of these cases from the call lists [23], however, hospitals and primary health care computer systems are not fully integrated, and family physicians do not always have access to information to identify those situations. During the period under review, 56% of referrals were cancelled due to this reason [31]. The same study also refers to the overloading of ophthalmology services with the dispensing of orthoptic technicians for screening. The authors conclude that, the screening programme relevance and advantage to public health is evident. However, they highlight that at a time when involvement in the programme represents an increased effort for ophthalmology services, it is important to optimise all steps of the process [31].

Hire orthoptic technicians exclusively for the screening programme could lighten the effort of hospital services, however, for that to happened, it is necessary to ensure an increased number of university positions in courses for orthoptic technicians [38]. On the other hand, optometrists claim for a more relevant role in DR screening planning and implementation [46]. In this context, ARS LTV is already hiring optometrists for primary health care units [38]. In England, this solution is implemented in a broader way. There are some regions where retinographies are carried out at high-street optometrists with cooperation protocols [17, 30, 47]. However, studies show that, in some of those areas, there are problems with access due to long waiting lists. So, uptake rates have not been found to be higher for those accessing screening services via high-street optometrists, despite this modality of screening being thought to offer increased proximity to the patients and appointment flexibility [18, 19, 29]. In Spain and in Mexico, retinographies and the first interpretation of the test are performed by family nurses or physicians. Then, there is a second valuation by the ophthalmologist, who knows the previous diagnosis and sends his opinion to primary care [17].

Computer systems are also important in the screening process: maintaining and sharing disease registers across different agents, management of patient records; automatic call/recall routines, electronic image transfer, and programme monitoring, are some aspects where new technologies have a critic role [16, 47].

The other major problem reported by CHP is the high burden that image grading and treatment of positive cases represents for hospitals ophthalmology services [31, 51]. Portugal may have about 1 million people with diabetes, of whom 700,000 diagnosed and on medical treatment and who should be consulted annually according to the DGS criteria. According to the Portuguese Ophthalmology Society, each of the 988 Portuguese ophthalmologists (2014 data) would have to observe about 708/each year, an infeasible number in terms of logistics specialty requirements. Moreover, only 422 of the 988 Portuguese ophthalmologists works in the SNS [53]. Automated grading software can decrease the cost of screening and reduce the amount of work for retinal grading ophthalmologists [8, 19, 28, 52]. Studies suggest it has an acceptable level of accuracy [19, 43, 44, 48, 49], and, besides the two Portuguese regions (ARSC and ARSLVT), it is already implemented throughout Scotland, in parts of Spain, Denmark and Hungary [16, 47, 50].

Mobile units using non-mydriatic cameras, may have an important role in increasing rates of screening attendance [47], another of the constrains mentioned by ARS.

In most ARS it has not been possible to have an annual frequency of generalised screening [51]. The implementation of screening programmes with extended intervals (more than 12 months between tests) may, in fact, be an option to free up resources and provide better care, but there are some concerns around this subject [20]. Actually, there are three determining factors when considering the use of extended screening intervals: the control of the diabetes, the sensitivity of the screening test and the adherence rate [20]. If the first two are objective and easy to quantify, the third factor may have more complex implications. Even if the rate of adherence of a certain population is high, it is possible that by increasing the interval between screenings, the message that the test is not important is being involuntarily transmitted, which can in the medium-term lead to a decrease of population adherence and consequently making the use of extended intervals a risky option [20]. Currently in Europe, the implementation of screening programmes with intervals of more than 1 year between calls is already quite frequent. However, some countries have adopted this measure in conjunction with the increased frequency of screening for diabetics identified as high risk (usually with calls every 6 months) [11, 47, 50]. On the other hand, the results of these options are not completely consistent. In Denmark and Finland there are no reports of problems associated with the increase of screening intervals, while in Sweden, the adherence rate has dropped significantly after the adoption of this measure (although the cause-and-effect relationship has not been fully proven) [47].

The actual practice in other countries shows that the medium- and long-term effect of rigorous screening implementation is effective [21, 47]. The United Kingdom began in the 1960s to screen diabetic retinopathy nationally and transversally [24]. It is concluded that in the 2009–10 biennium, for the first time, diabetic retinopathy was not the first cause for the attestation of incapacity for visual blindness of working age in England and Wales, 40 years after the implementation of the screening [24, 47]. Therefore, it is not expected that the implementation of public policies on diabetes and diabetic retinopathy lead to visible results in 3 or 4 years, but those results should appear in the medium and long term [21].

## Conclusions

This study allowed the analysis of the diabetic retinopathy screenings implemented in mainland Portugal. There was some difficulty in collecting uniform data since there are different degrees of implementation, methodologies, and monitoring in the five ARS. However, this analysis allows to assess the differences, detect constraints, and identify possible solutions and improvements.

The main conclusion is that access to diabetic retinopathy screening remains remarkably variable in Portugal and needs urgent attention. Due to its importance DR screening should be a public health priority, and governments should ensure adequate funding to population-based programmes. National guidelines should also precise the role of the different intervenient, and politic measures should be created to guarantee the involvement of all parts.

Even though characteristics of effective screening programmes (adequate sensitivity and specificity, a convenient method for the patient, proximity strategies) were found in Portuguese screening programmes, which could be pointing towards promising outcomes, we notice lots of room for improvement. With a continued effort, hopefully, in a few years there will be a national, standardised, population-based, DR screening programme.

The findings of this research could be very useful for other Countries with similar socio-political characteristics.

## Supplementary Information

**Additional file 1.** PRISMA 2009 Checklist.

**Additional file 2.** Overview of the Portuguese governmental and non-governmental health organizations, with a relevant role on DR Screening.

**Additional file 3.** Quality assessment of the selected scientific papers.

## Data Availability

Abstracted data collected and analysed during this study and described in this systematic review will be available from the corresponding author upon request.

## Abbreviations

ACES: Primary Health Centre Clusters
APDP: Association for the Protection of Diabetics of Portugal
ARS: Regional Health Administrations
ARS Alentejo: Alentejo Regional Health Administration
ARS Algarve: Algarve Regional Health Administration
ARSC: Central Region Health Administration
ARSLVT: Lisbon and Tagus Valley Regional Health Administration
ARSN: North Regional Health Administration
CHP: Hospital Centre of Oporto
DGS: Portuguese General Health Department
DM: Diabetes Mellitus
DR: Diabetic Retinopathy
PRISMA: Preferred Reporting Items for Systematic Reviews and Meta-Analysis
SNS: National Health System
RETINODIAB: Diabetic Retinopathy Screening Service for Lisbon and Tagus Valley
