# Supplementary material for: Five regions, five retinopathy screening programmes: a systematic review of how Portugal addresses the challenge
Source: BMC Health Serv Res. 2021 Jul 30;21:756. doi: 10.1186/s12913-021-06776-8 (PMC8325279; doi:10.1186/s12913-021-06776-8)
Supplement: Supplementary file 2 — Additional file 2. Overview of the Portuguese governmental and non-governmental health organizations, with a relevant role on DR Screening. [file 12913_2021_6776_MOESM2_ESM.docx]

**Additional file 2. Overview of the Portuguese governmental and non-governmental health organizations, with a relevant role on DR Screening.**

Briefly, the public healthcare system in Portugal is delivered through the Portuguese National Health Service (SNS). The Portuguese SNS comprehends institutions within the government direct and indirect administration. Among those institutions, we will focus only in the ones with a relevant role in DR screening (Figure 1 illustrates their hierarchical organization) (1).

SNS is managed by the Central Administration of the Health System (ACSS), and delivered by five Regional Health Administrations (ARS North, Central, Lisbon and Tagus Valley, Alentejo and Algarve). SNS covers Primary Health Care (Primary Health Centers), and Secondary Care (hospitals and specialist units) (1) (3).

Primary Health Centers are associated in Health Center Clusters (Agrupamentos de Centros de Saúde - ACES) with administrative autonomy, decentralized from ARS but subjected to their directive power (1) (2) (3) .

With regard to hospital institutions, the articulation with guardianship (Regional / central Administration) is currently materialized through a negotiation process based on the link between the allocated funding and the results expected (3).

The management contract consists of duties and obligations translated in to physical and quality goals and is an important tool because it allows to monitor the performance of the hospital service, so that necessary interventions can be performed (3).

The contract with the hospitals is supervised by ACSS, which has the strategic responsibility to make the contracting process compatible with the health policy objectives (1) (3). ARS have the responsibility to operationalize the whole process, from the elaboration of contracts, to the monitoring, evaluation, and negotiation of the incentive system (1) (2) (4).

The General Health Department (Direção Geral de Saúde - DGS) is a government institution, with a vital role on the organization and monitoring of population-based screenings. DGS has the mission of regulate, guide and coordinate activities of health promotion and disease prevention, define the technical conditions for adequate health care, as well as ensuring the elaboration and execution of the National Health Plan (4).

Figure 1 – Portuguese SNS Organization


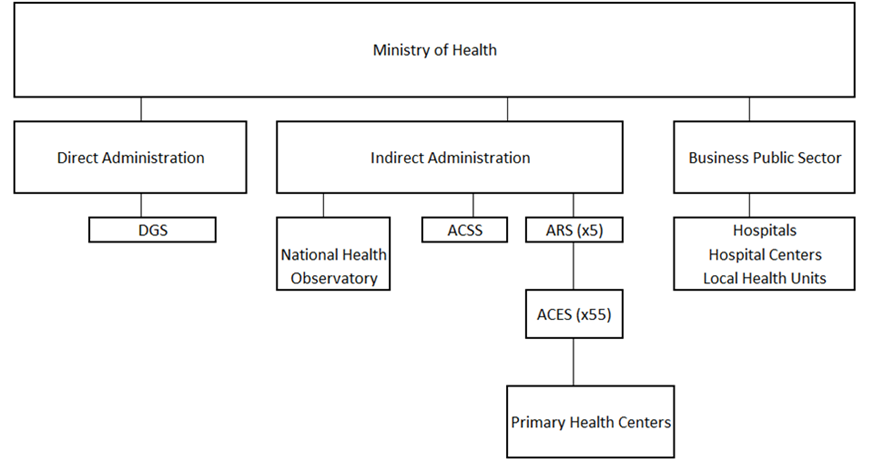


The National Institute of Health Doctor Ricardo Jorge (ONS) also has an important role on RD Screening. ONS is a public body integrated in the indirect administration of the State, endowed with scientific, technical, administrative, financial and proprietary autonomy. It develops a triple mission as state laboratory in the health sector, national reference laboratory and national health observatory (5).

Aside from the governmental organizations involved in DR screening programmes, there are also two non-governmental organizations with very important roles: The National Diabetes Observatory (Observatório Nacional da Diabetes - OND) and the Portuguese Diabetes Association (Associação Protetora dos Diabéticos de Portugal -APDP). OND is responsible for collecting, validating, generating and disseminating reliable and scientifically credible information on Diabetes in Portugal. APDP is the world’s oldest diabetes association and a senior member of the International Diabetes Federation. It is a non-governmental institution, which aims to improve the quality of life of people with diabetes (6).

# **References**

x

| 1. | Portuguese Ministry of health. Decreto-Lei nº 22/2012. 2012.. |
| --- | --- |
| 2. | National Health System - SNS. [Online]. [cited 2019 11 2. Available from: <https://www.sns.gov.pt/>. |
| 3. | Central Administration of the Health System - ACSS. Rede nacional de especialidade hospitalar e de referenciação: oftalmologia. ; 2017. |
| 4. | General Health Department. [Online]. [cited 2019 12 13. Available from: <https://www.dgs.pt/>. |
| 5. | National Institute Of Health Doctor Ricardo Jorge - INS. [Online]. [cited 2019 10 18. Available from: <http://www.insa.min-saude.pt/category/areas-de-atuacao/>. |
| 6. | Portuguese Society of Diabetology. [Online]. [cited 2019 10 25. Available from: <https://www.spd.pt/index.php/observatrio-mainmenu-330>. |

x
