# Supplementary material for: Five regions, five retinopathy screening programmes: a systematic review of how Portugal addresses the challenge
Source: BMC Health Serv Res. 2021 Jul 30;21:756. doi: 10.1186/s12913-021-06776-8 (PMC8325279; doi:10.1186/s12913-021-06776-8)
Supplement: Supplementary file 3 — Additional file 3. Quality assessment of the selected scientific papers. [file 12913_2021_6776_MOESM3_ESM.docx]

**Additional file 3. Quality assessment of the selected scientific papers.**

| **Paper** | **Ref** | **Year** | **Journal** | **PQ01** | **PQ02** | **PQ03** | **PQ04** | **PQ05** | **PQ06** | **PQ07** | **PQ08** | **PQ09** | **Total** |
| --- | --- | --- | --- | --- | --- | --- | --- | --- | --- | --- | --- | --- | --- |
| Epidemiology of diabetic retinopathy and macular edema: a systematic review. | 1 | 2004 | Eye | 0 | 1 | 1 | 0 | 0 | 0 | 0 | 0 | 0 | 2 |
| Global Prevalence of Diabetes: Estimates for the year 2000 and projections for 2030. | 2 | 2004 | Diabetes Care | 0 | 1 | 1 | 0 | 0 | 0 | 0 | 0 | 0 | 2 |
| Global estimates of undiagnosed diabetes in adults. | 3 | 2014 | Diabetes Research and Clinical Practice | 0 | 1 | 1 | 0 | 0 | 0 | 0 | 0 | 0 | 2 |
| Diabetes in Europe: An update. | 5 | 2014 | Diabetes Research and Clinical Practice | 0 | 1 | 1 | 0 | 0 | 0,5 | 0 | 0 | 0 | 2,5 |
| Burden of illness of diabetic macular edema: literature review. | 6 | 2010 | Current Medical Research & Opinion | 0,5 | 1 | 1 | 0,5 | 0,5 | 0 | 0 | 0,5 | 0 | 4 |
| Major automatic diabetic retinopathy screening systems and related supporting algorithms: a review. | 7 | 2019 | Machine Vision and Applications | 0,5 | 1 | 1 | 0,5 | 0,5 | 0,5 | 0,5 | 0,5 | 0 | 5 |
| Prevalence of diabetic retinopathy in India: The All India Ophthalmological Society Diabetic Retinopathy Eye Screening Study 2014. | 8 | 2015 | Indian Journal of Ophthalmology | 0,5 | 1 | 1 | 1 | 0 | 0,5 | 0,5 | 0,5 | 0 | 5 |
| Retinal Imaging Techniques for Diabetic Retinopathy Screening. | 9 | 2016 | Journal of Diabetes Science and Technology | 0,5 | 1 | 1 | 0,5 | 0,5 | 0,5 | 0,5 | 0,5 | 0 | 5 |
| Epidemiology of diabetes and complications among adults in the Republic of Ireland 1998-2015: a systematic review and meta-analysis. | 10 | 2016 | BMC Public Health | 0 | 1 | 1 | 0 | 0 | 0 | 0 | 0 | 0 | 2 |
| Epidemiology of diabetic retinopathy, diabetic macular edema and related vision loss. | 11 | 2015 | Eye and Vision | 0 | 1 | 1 | 0,5 | 0,5 | 0 | 0 | 0 | 0 | 3 |
| Screening intervals for diabetic retinopathy and incidence of visual loss: a systematic review. | 12 | 2013 | DIABETIC  Medicine | 0,5 | 1 | 1 | 0,5 | 0,5 | 0,5 | 0,5 | 0,5 | 0 | 5 |
| Evidence-Based Medicine of Screening of Diabetic Retinopathy among Type 2 Diabetes: A Clinical Overview. | 13 | 2015 | Health | 0,5 | 1 | 1 | 0,5 | 0,5 | 1 | 1 | 1 | 0 | 6,5 |
| Prevalence of Diabetic Retinopathy in Various Ethnic Groups: A Worldwide Perspective. | 14 | 2012 | Survey of ophthalmology | 0 | 1 | 1 | 0,5 | 0 | 0,5 | 0 | 0 | 0 | 3 |
| Frequency of diabetic retinopathy and associated risk factors in Khartoum, Sudan: population-based study. | 15 | 2017 | International Journal of Ophthalmology | 1 | 1 | 1 | 1 | 0 | 0 | 0 | 0 | 0 | 4 |
| Review of diabetic retinopathy screening methods programmes adopted in different parts of the world. | 17 | 2016 | European Ophthalmic Review | 1 | 1 | 1 | 1 | 0 | 0,5 | 0,5 | 0,5 | 0 | 5,5 |
| Automated diabetic retinopathy imaging in Indian eyes: A pilot study. | 19 | 2014 | Indian Journal of Ophthalmology | 0,5 | 1 | 1 | 0,5 | 0,5 | 0,5 | 0,5 | 0,5 | 0 | 5 |
| Diabetic retinopathy: global prevalence, major risk factors, screening practices and public health challenges: a review. | 20 | 2016 | Clinical and Experimental Ophthalmology | 0,5 | 1 | 1 | 1 | 0,5 | 1 | 1 | 1 | 0 | 7 |
| Diabetic Retinopathy Screening: Progress or Lack of Progress. | 21 | 2012 | Ophthalmology Research | 0,5 | 1 | 1 | 0,5 | 0 | 0,5 | 0,5 | 0,5 | 0 | 4,5 |
| Automated detection of Diabetic Retinopathy in Three European Populations. | 28 | 2016 | Journal of Clinical & Experimental Ophthalmology | 0,5 | 1 | 1 | 0,5 | 0,5 | 0,5 | 0,5 | 0,5 | 0 | 5 |
| First 5 years of Implementation of Diabetic Screening Program in Centro Hospitalar do Porto. | 31 | 2017 | Revista Brasileira Oftalmologia, | 1 | 1 | 1 | 1 | 0,5 | 1 | 1 | 1 | 1 | 8,5 |
| SCREEN-DR: Collaborative platform for diabetic retinopathy. | 42 | 2018 | International journal of medical informatics | 1 | 1 | 1 | 1 | 0,5 | 0,5 | 0,5 | 0,5 | 0,5 | 6,5 |
| Screening for Diabetic Retinopathy in the Central Region of Portugal. Added Value of Automated 'Disease/No Disease' Grading. | 43 | 2014 | Ophthalmologica | 1 | 1 | 1 | 1 | 0,5 | 0,5 | 0,5 | 0,5 | 0,5 | 6,5 |
| Improved automated screening of diabetic retinopathy. | 44 | 2011 | Ophthalmologica | 1 | 1 | 1 | 1 | 0,5 | 0,5 | 0,5 | 0,5 | 0,5 | 6,5 |
| First Diabetic Retinopathy Prevalence Study in Portugal, the RETINODIAB Study - Evaluation of the Screening Programme for Lisbon and Tagus Valley Region. | 45 | 2015 | American Academy of Ophthalmology | 1 | 1 | 1 | 1 | 0 | 0 | 0 | 0,5 | 1 | 5,5 |
| Automated Screening for Diabetic Retinopathy – A Systematic Review. | 49 | 2017 | Ophthalmic Research | 0,5 | 1 | 1 | 0,5 | 0,5 | 0,5 | 0,5 | 0,5 | 0 | 5 |
| Automated Retinal Image Analysis for Diabetic Retinopathy in Telemedicine. | 51 | 2015 | Current Diabetes Reports | 0,5 | 1 | 1 | 1 | 0,5 | 0,5 | 0,5 | 0,5 | 0,5 | 6 |
| Automated detection of diabetic retinopathy in retinal images. | 52 | 2016 | Indian Journal of Ophthalmology | 0,5 | 1 | 1 | 0,5 | 0,5 | 0,5 | 0,5 | 0,5 | 0 | 5 |
| Glycaemic threshold for diabetes-specific retinopathy among individuals from Saudi Arabia, Algeria and Portugal. | 54 | 2013 | Diabetes Research and Clinical Practice | 1 | 1 | 1 | 1 | 0,5 | 0 | 0 | 0 | 0 | 4,5 |
